# Efficacy of Different Energy Levels Used in Focused and Radial Extracorporeal Shockwave Therapy in the Treatment of Plantar Fasciitis: A Meta-Analysis of Randomized Placebo-Controlled Trials

**DOI:** 10.3390/jcm8091497

**Published:** 2019-09-19

**Authors:** Ying-Chun Wang, Shu-Jung Chen, Peng-Ju Huang, Hsuan-Ti Huang, Yuh-Min Cheng, Chia-Lung Shih

**Affiliations:** 1 Department of Orthopedics, Kaohsiung Municipal Ta-Tung Hospital, Kaohsiung Medical University, Kaohsiung 801, Taiwan; 2 Department of Orthopedics, Kaohsiung Medical University Hospital, Kaohsiung Medical University, Kaohsiung 807, Taiwan; 3 Department of Orthopedics, College of Medicine, Kaohsiung Medical University, Kaohsiung 807, Taiwan; 4 Department of Orthopedics, Ministry of Health and Welfare Pingtung Hospital, Pingtung 900, Taiwan

**Keywords:** extracorporeal shockwave therapy, plantar fasciitis, treatment success rate, visual analog scale

## Abstract

The objective of this study was to assess the efficacy of different energy levels used in extracorporeal shockwave therapy (ESWT) in the treatment of plantar fasciitis using a systematic review and meta-analysis. We searched PubMed, Embase, and Cochrane library, from inception to March 2019 for randomized controlled trials that compared ESWT with placebo in patients with plantar fasciitis. The risk of bias for selected articles was assessed based on the Cochrane Handbook Systematic Review of Interventions. The pooled data were estimated by the mean difference or odds ratio. The meta-analysis showed that the high-energy ESWT group had a better success rate than the control group only at a three-month follow-up, but no significant difference between groups was observed for the other follow-up visits (1 and 12 months). In addition, no significant differences in visual analog scale (VAS) scores between groups were observed for all the follow-up visits (one-month and three-month). On the contrary, the medium-energy ESWT group had significantly better success rates than the control group for all the follow-up visits (3, 6, and 12 months). In addition, the medium-energy ESWT group had significant improvement in VAS scores compared with the control group for all the follow-up visits (1, 3, 6, and 12 months) after removing the extreme values. The low-energy ESWT group had significant improvement in VAS scores compared with the control group for all the follow-up visits (3 and 12 months). Otherwise, focused ESWT seems to be more effective than radial ESWT when compared with the control group. Use of local anesthesia can reduce the efficacy of low- and high-energy ESWTs. Our meta-analysis suggested that medium-energy ESWT in the treatment of plantar fasciitis was more effective than the control group. A limited number of trials related to low- and high-energy ESWTs were included in our meta-analysis. More research is required to confirm the efficacy of low- and high-energy ESWTs in future studies.

## 1. Introduction

Plantar fasciitis is a common foot disease and it causes inferior heel pain [1]. Approximately 10% of the U.S. population have suffered from this disease, which occurs particularly in woman at the age of 40–70 years [2]. About 10% to 20% of patients would develop chronic pain [1]. The most serious heel pain usually occurs after a rest period or the first steps taken in the morning [1]. Several risk factors could lead to plantar fasciitis, such as obesity, ankle dorsiflexion, and heel spurs [2]. People with a body mass index (BMI) of >30 kg/m^2^ had a higher risk of plantar fasciitis (odds ratio: 5.6) compared with those with a BMI ≤ 25 kg/m^2^ [3]. People with <0° of ankle dorsiflexion showed a higher risk of plantar fasciitis (odds ratio: 23.3) compared with those with >10° of ankle dorsiflexion [3]. Approximately 50% of patients diagnosed with plantar fasciitis were observed to have heel spurs [4]. Plantar fasciitis pain has a negative impact on health-related quality of life in patients [5,6]. Females have a worse quality of life with regard to footwear, foot function, foot pain, and general foot health than males [5]. Surgical treatment of plantar fasciitis would be the last choice of interventions. To avoid surgical treatment, several non-surgical treatments have been developed to treat this disease, such as physical therapy, nonsteroidal anti-inflammatory drugs, corticosteroid injections, and shoe modification [7,8,9,10].

Extracorporeal shockwave therapy (ESWT) in the treatment for a number of musculoskeletal diseases has been investigated since the early 1990s, such as chronic plantar fasciitis, painful tendinosis, and calcific tendinosis of the shoulder [11]. Shockwaves are acoustic waves and produce a sudden rise in pressure. The energy flux can be concentrated on a small area. The specific mechanism of ESWT remains unclear, and it was speculated that ESWT could induce excitability of the axon and destroy unmyelinated sensory fibers to produce an analgesic effect [12]. ESWT causes fewer adverse events, and most patients can recover from these events after a few days [13]. The cost of ESWT is relatively low compared with surgery; thus, ESWT has been generally used to treat plantar fasciitis [14]. The two different types of ESWT, radial and focused ESWTs, are widely used to treat plantar fasciitis [15]. Shockwaves that are generated from these two types of ESWT have dramatic differences in physical characteristics. Radial ESWT is pneumatic waves which are generated by an air compressor through a tube on the end of an applicator. The projectile hits the applicator and the generated pressure wave is transmitted into the body by the applicator. For focused ESWT, the shockwaves can be generated by three methods, including electromagnetic, electrohydraulic, and piezoelectric [16]. The shockwaves are generated in water for all the three methods. Without any loss in energy, the shockwaves are better transferred into the body.

To achieve the best improvement in pain relief of plantar fasciitis, the energy level of ESWT has been adjusted to the maximum level that patients can tolerate the pain induced by ESWT. According to a previous study [17], ESWT can be separated to low- (energy flux density <0.1 mJ/mm^2^), medium- (energy flux density: 0.1–0.2 mJ/mm^2^), and high-energy (energy flux density ≥0.2 mJ/mm^2^) treatments regardless of the types of shockwave generators, and these three energy levels have been widely adopted to treat plantar fasciitis [18]. Although previous meta-analyses have investigated the efficacy of different energy levels used in ESWT, the results are not consistent [15,18,19,20]. Some factors which could affect the efficacy of ESWT have not been considered in all the previous meta-analysis [15,18,19,20]. For example, the efficacy of ESWT could be varied with different follow-up periods but has not been considered in all the previous meta-analyses. The efficacy between radial and focused ESWTs is reported to be different in only one previous meta-analysis [15]. Local anesthesia is reported to reduce the efficacy when using ESWT in the treatment of plantar fasciitis [21] but this effect on clinical outcomes has not been investigated using meta-analysis. It is important to clear the efficacy of ESWT with different energy levels and consider the effect of these factors on clinical outcomes.

The objective of this study was to use a systematic review and meta-analysis for assessing the efficacy of different energy levels used in ESWT to treat plantar fasciitis. Moreover, the factors, including different follow-ups, different types of ESWT, and local anesthesia that could affect the clinical outcomes were investigated in this meta-analysis.

## 2. Materials and Methods

### 2.1. Search Strategy

We followed the guidelines by the Preferred Reporting Items for Systematic Reviews and Meta-Analyses to conduct this study [22]. We adhered to PRISMA checklist for this study (Appendix A). We searched relevant articles from three electronic databases, including the Embase, Pubmed, and Cochrane library, from inception to March 2019 for randomized controlled trials (RCTs) that compared ESWT with placebo-controlled group in patients with plantar fasciitis. Boolean logic with search terms was used to search articles from the databases, and the following specifics were used to search articles: (“Plantar fasciitis” OR “plantar fasciopathy” OR “heel spur syndrome”) AND (”shockwave“ OR ”shock wave“). To identify additional eligible articles that may not be found in the three databases, references cited by the relevant articles or reviews were also manually searched. Ethical approval was not required in this study because only the data from previous published studies were adopted in the meta-analysis.

### 2.2. Inclusion and Exclusion Criteria

The inclusion criteria for the articles were as follows: (1) RCTs focusing on comparing ESWT and placebo-controlled groups; (2) patients with plantar fasciitis for adults; (3) the outcomes of interest that was reported, including visual analog scale (VAS) and treatment success rate; (4) articles that were originally written in English; and (5) a fixed energy level of ESWT regardless whether ESWT was generated by electrohydraulic, electromagnetic, piezoelectric, or air compressor methods. The exclusion criteria were as follows: (1) Cohort studies, retrospective studies, and non-randomized studies; (2) letters, editorials, case reports, review articles, and conference abstracts; (3) animal studies; and (4) inability to extract the outcome measures (VAS scores and treatment success rates). Because the follow-up duration was varied among articles, the follow-up times were grouped into 1 month (3–6 weeks), 3 months, 6 months, and 12 months (12–13 months) for assessment of the efficacy of ESWT for different durations.

### 2.3. Type of Outcomes

The success treatment rate and VAS score were the two outcome measures that were adopted to assess the efficacy of low-, medium-, and high-energy ESWTs in our meta-analysis. The definition of successful treatments was varied among articles, such as 60% improvement in pain for at least 2/3 of pain measurements, 60% decrease of VAS, 60% improvement in pain from baseline, 50% reduction in morning pain, and 50% improvement of morning pain from baseline. A previous study has confirmed that ≥50% decrease of VAS score can be defined as successful pain management [23]. The VAS score is widely used to measure a patient’s pain level [24]. The score is self-reported measures of symptoms and ranged from 0 to 10, where 0 indicates no pain and 10 indicates the worst pain [24].

### 2.4. Data Extraction 

Two authors (Y.-C.W. and C.-L.S.) independently evaluated all potential articles based on the aforementioned criteria. First, title and abstract of each article was reviewed to exclude articles based on the exclusion criteria. Second, the remaining articles were reviewed via full-text analysis. Any disagreements were discussed by the two authors until consensus. Data were independently extracted by the two authors, including author’s name, publication year, study design, use of local anesthesia, number of patients, mean age, intervention details, energy flux density, follow-up times, extracted outcomes, and definition of treatment success.

### 2.5. Quality Assessment

The risk of bias for each selected article was assessed independently by the two authors based on a template from the Cochrane Handbook Systematic Review of Interventions [25]. The items of assessment included random sequence generation, blinding of outcome assessment, allocation concealment, incomplete outcome data, evaluation of reporting bias, and other types of bias. Each item was assessed using three grades, including low risk, unclear risk, and high risk. Any disagreements were discussed by the two authors until consensus was reached.

### 2.6. Statistical Analysis

The included articles were divided into 3 groups based on the treatment intensity of ESWT regardless of the types of shockwave generators: Low-energy (energy flux density <0.10 mJ/mm^2^), medium-energy (energy flux density: 0.10–0.20 mJ/mm^2^), and high-energy (energy flux density ≥0.2 mJ/mm^2^) [18]. Each group was divided into subgroups according to the follow-up duration (1, 3, 6, and 12 months), and these subgroups were analyzed respectively. Meta-analysis and forest plotting were conducted using the Review Manager 5.3 software (Cochrane Collaboration, Oxford, UK). A *p*-value smaller than 0.05 was considered significant for all the tests. The effect sizes for outcome measures between ESWT and control groups for the selected articles were estimated. For continuous data, the effect size referred to the mean difference (MD) with 95% confidence interval (CI). For dichotomous data, the effect size was calculated using odds ratio (OR) with 95% CI. Heterogeneity among articles was evaluated using the chi-squared test and *I*^2^ statistic, while a *p*-value of the chi-squared test ≥0.05 indicated no significant heterogeneity and <0.05 indicated significance. The *I*^2^ statistic was used to evaluate the level of heterogeneity. Heterogeneity was considered low, moderate, high, or very high when *I*^2^ was <25%, 25%–50%, 50%–75%, or >75%, respectively [26]. Two models, a fixed-effect and a random-effect model, could be chosen for meta-analysis according to the *p*-value of chi-square test and the level of heterogeneity. A fixed-effect model was used when the *p*-value was ≥0.05 and *I*^2^ < 50%; otherwise, a random-effect model was used when the *p*-value was <0.05 and *I*^2^ > 50%. To determine the robust of results from meta-analysis, a sensitivity analysis was performed. In the sensitivity analysis procedure, the meta-analysis was repeated with an altered dataset.

## 3. Results

### 3.1. Study Characteristics

**Figure 1:**
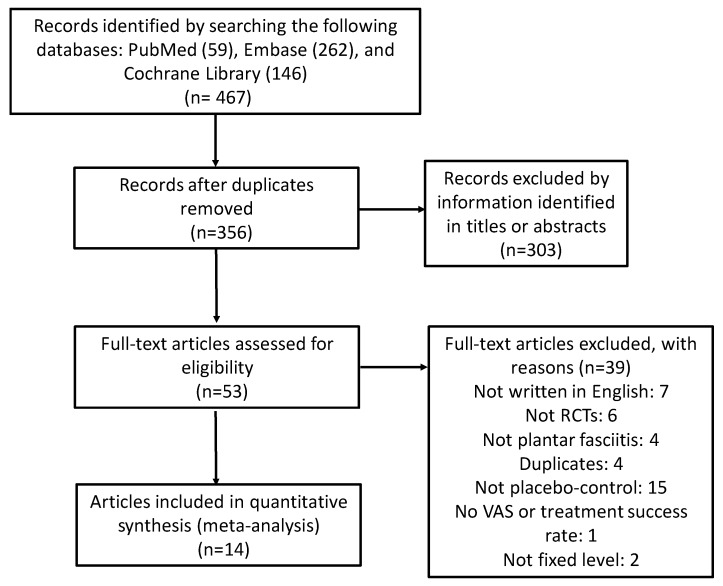
Flow diagram of selection process for relevant article identification.

The selection process of relevant articles is described below (Figure 1). A total of 467 articles were initially retrieved from the three databases. After removal of duplicates, the remaining articles numbered 356. Fifty-three articles were considered as potential articles after reviewing titles and abstracts. After full-text review, 14 articles published between 1996 and 2019 met the inclusion criteria for this study [13,27,28,29,30,31,32,33,34,35,36,37,38,39]. These articles were all randomized placebo-controlled trials, and there were 868 patients in the ESWT groups and 846 in the placebo-controlled groups (Table 1). Patient mean ages ranged from 40.0 to 58.9 years, trial duration ranged from three weeks to 13 months, and the energy flux density used in the ESWT groups ranged from 0.08 to 0.36 mJ/mm^2^.

### 3.2. Quality Assessment

**Figure 2:**
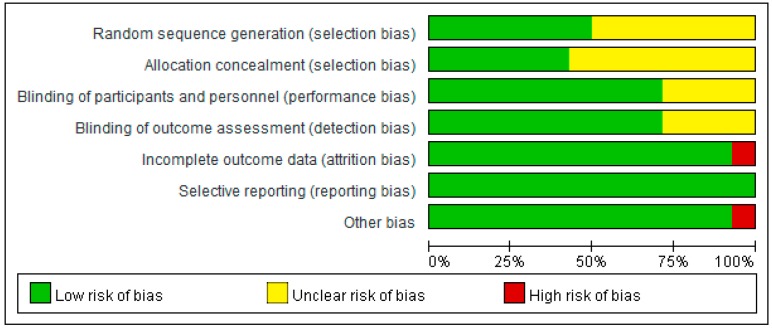
Summary of risk of bias for all the included articles.

**Figure 3:**
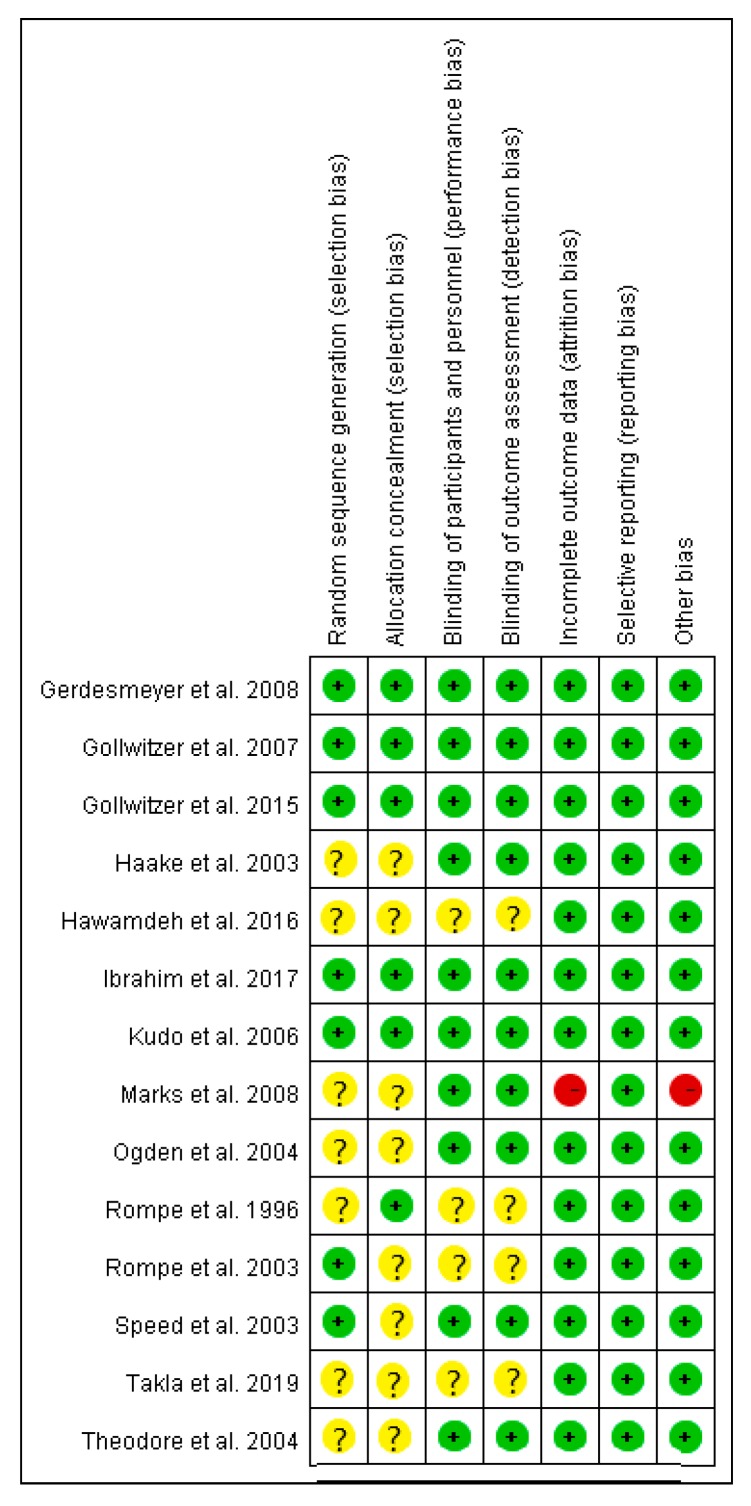
Risk of bias of each included article assessed by the two authors for each risk item. The symbol “+” indicates low risk, the symbol “?” indicates unclear risk, and the symbol “−“ indicates high risk.

The risk of bias of all the included articles was evaluated (Figure 2 and Figure 3). All the 14 included articles were randomized trials. Seven mentioned random sequence generation and six mentioned allocation concealment. Eleven articles were double-blinded trials, three were single-blinded, and one did not mention double- or single-blinding in the study design. The risk of incomplete outcome data was low for 13 articles. All articles had low risk of selective reporting. The other type of risk was low for 13 articles, and one article had a small sample size.

### 3.3. Meta-Analysis

#### 3.3.1. Different Energy Levels of ESWT regardless of the Types of Shockwave Generators 

##### Success Rate

**Figure 4:**
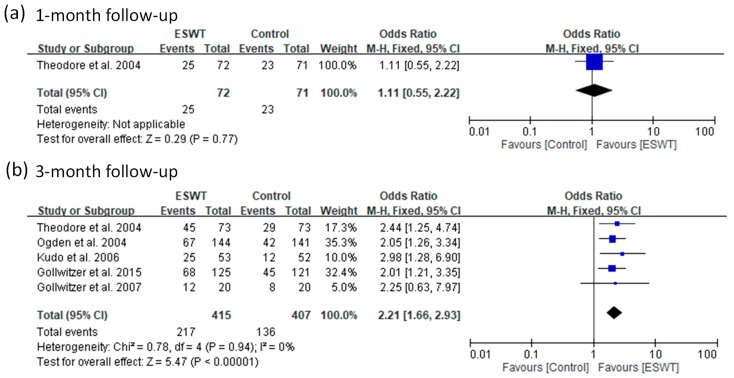
Forest plots of treatment success rates in high-energy extracorporeal shockwave therapy (ESWT) and placebo-controlled groups at 1-month (**a**) and 3-month (**b**) follow-ups.

Nine of the 14 included articles reported a treatment success rate. There were 705 patients in the ESWT groups and 687 in the placebo-controlled groups. For the high-energy group, they reported a treatment success rate for two follow-up visits (one and three months). The pooled data showed no significant heterogeneity at three-month follow-up (*p*-value of chi-square = 0.94 and *I*^2^ = 0%) (Figure 4). Heterogeneity could not be assessed for the other subgroup because this only had one article that reported a success rate. The ESWT group had a better success rate than the control group at three-month follow-up (fixed-effect model, five trials, OR = 2.21, 95% CI = 1.66 to 2.93, *p* < 0.00001) and the other follow-up visit (one-month) showed no significant differences between groups (Figure 4).

**Figure 5:**
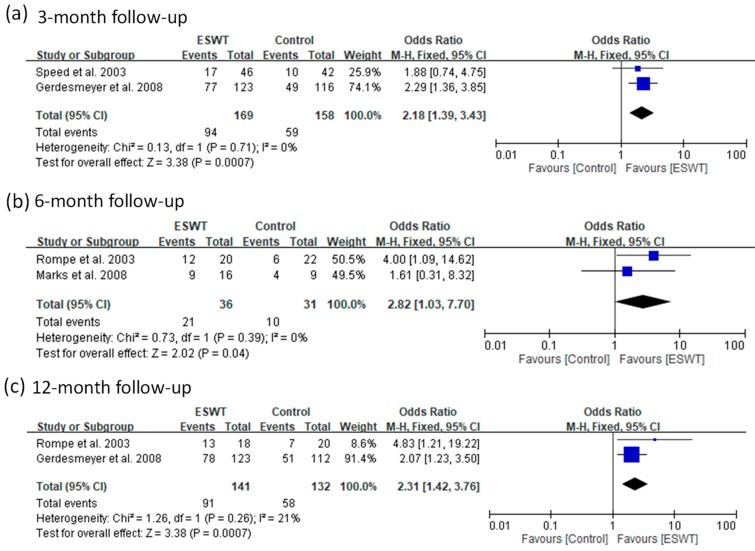
Forest plots of treatment success rates in medium-energy ESWT and placebo-controlled groups at 3-month (**a**), 6-month (**b**), and 12-month (**c**) follow-ups.

For the medium-energy group, four articles reported a success rate for three follow-up visits (3, 6, and 12 months). The ESWT group had a better success rate compared with the control group from three-month to 12-month follow-ups (fixed-effect model, two trials, OR = 2.18, 95% CI = 1.39 to 3.43, *p* = 0.0007 at three-month; fixed-effect model, two trials, OR = 2.82, 95% CI = 1.03 to 7.70, *p* = 0.04 at six-month; fixed-effect model, two trials, OR = 2.31, 95% CI = 1.42 to 3.76, *p* = 0.0007 at 12-month) (Figure 5).

**Figure 6:**
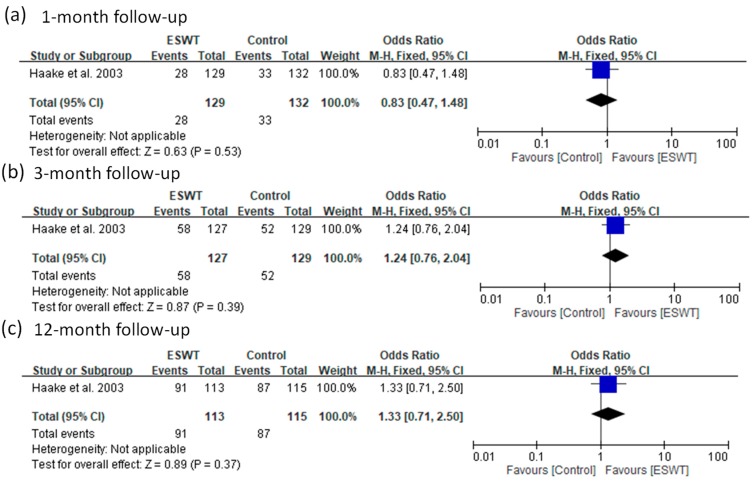
Forest plots of treatment success rates in low-energy ESWT and placebo-controlled groups at 1-month (**a**), 3-month (**b**), and 12-month (**c**) follow-ups.

For the low-energy group, only one article reported a success rate at three follow-up visits (1, 3, and 12 months). There were no significant differences in success rates between the ESWT and control groups for all the follow-ups (fixed-effect model, one trial, OR = 0.83, 95% CI = 0.47 to 1.48, *p* = 0.53 at one-month; fixed-effect model, one trial, OR = 1.24, 95% CI = 0.76 to 2.04, *p* = 0.39 at three-month; fixed-effect model, one trial, OR = 1.33, 95% CI = 0.71 to 2.50, *p* = 0.37 at 12-month) (Figure 6).

##### VAS Score

**Figure 7:**
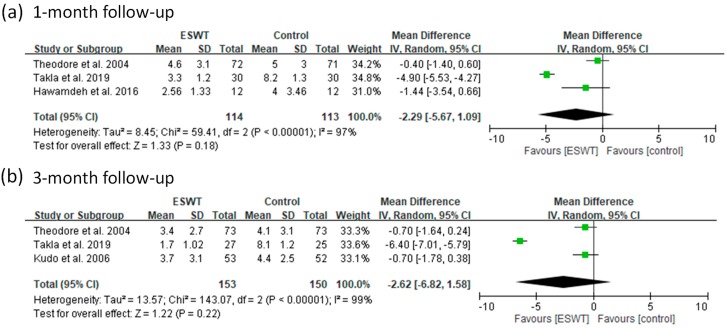
Forest plots of visual analog scale (VAS) scores in high-energy ESWT and placebo-controlled groups at 1-month (**a**) and 3-month (**b**) follow-ups.

Nine of the 14 included articles reported VAS scores to assess a patient’s pain level in different follow-up periods. There were 331 patients in the ESWT groups and 327 in the placebo-controlled groups. For the high-energy group, these articles reported VAS scores at one-month and three-month follow-ups. The pooled data showed significant heterogeneity (*p*-value of chi-square <0.000001 and *I*^2^ = 97% to 99%) for all the follow-up visits (Figure 7). There were no significant differences between the ESWT and control groups for all the follow-up visits (random-effect model, three trials, MD = −2.29, 95% CI = −5.67 to 1.09, *p* = 0.18 at one-month; random-effect model, three trials, MD = −2.62, 95% CI = −6.82 to 1.58, *p* = 0.22 at three-month) (Figure 7).

**Figure 8:**
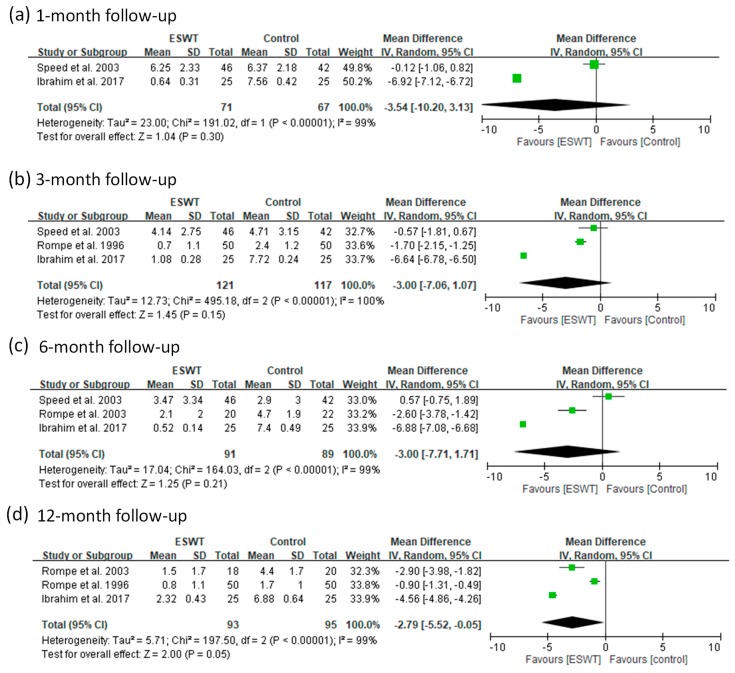
Forest plots of visual analog scale (VAS) scores in medium-energy ESWT and placebo-controlled groups at 1-month (**a**), 3-month (**b**), 6-month (**c**), and 12-month (**d**) follow-ups.

For the medium-energy group, these articles reported VAS scores for four follow-up visits (1, 3, 6, and 12 months). The pooled data showed significant heterogeneity (*p*-value of chi-square <0.000001 and *I*^2^ = 99% to 100%) for all the follow-up visits (Figure 8). The ESWT group had significant better improvement in VAS score than the control group only at 12-month follow-up (random-effect model, two trials, MD = −3.54, 95% CI = −10.20 to 3.13, *p* = 0.30 for one-month; random-effect model, three trials, MD=−3.00, 95% CI = −7.06 to 1.07, *p* = 0.15 at three-month; random-effect model, three trials, MD = −3.00, 95% CI = −7.71 to 1.71, *p* = 0.21 at six-month; random-effect model, three trials, MD = −2.79, 95% CI = −5.52 to −0.05, *p* = 0.05 at 12-month) (Figure 8). The sensitivity analyses for these subgroups were performed (Table S1). After removing the article [29], the ESWT group had greater improvement in VAS pain scores compared with the control group for one- to six-month follow-ups (fixed-effect model, one trial, MD = −6.92, 95% CI = −7.12 to −6.72, *p* < 0.00001 for one-month; fixed-effect model, one trial, MD = −6.64, 95% CI = −6.78 to −6.50, *p* < 0.00001 for three-month; random-effect model, two trials, MD = −4.78, 95% CI = −8.97 to -0.59, *p* = 0.03 for six-month) (Table S1 and Figure S1). The results indicate that the VAS scores reported by the article [29] seem to be extreme values and should be removed from our meta-analysis.

**Figure 9:**
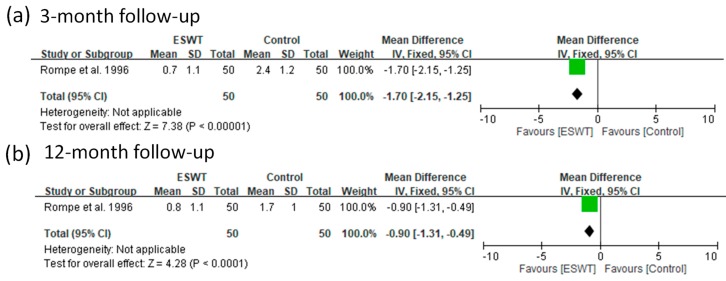
Forest plots of visual analog scale (VAS) scores in low-energy ESWT and placebo-controlled groups at 3-month (**a**) and 12-month (**b**) follow-ups.

For the low-energy group, we found that only one article reported VAS scores for two follow-up visits (3 and 12 months). The ESWT group had greater improvement in VAS pain scores compared with the control group for all the follow-ups (fixed-effect model, one trial, MD = −1.70, 95% CI = −2.15 to −1.25, *p* < 0.00001 for three-month; fixed-effect model, one trial, MD = −0.90, 95% CI = −1.31 to −0.49, *p* < 0.0001 for 12-month) (Figure 9).

#### 3.3.2. Radial and Focused ESWTs 

##### Success Rate

For the medium-energy level, two articles used radial ESWT to treat plantar fasciitis and two articles used focused ESWT. The pooled data showed no significant heterogeneity (*p*-value of chi-square >0.05 and *I*^2^ = 0%) at three–six months follow-ups (Figures S2 and S3). The radial ESWT group had a better success rate than the control group at 3–6 and 12 months follow-ups (fixed-effect model, two trials, OR = 2.22, 95% CI = 1.35 to 3.63, *p* = 0.002 for three–six months; fixed-effect model, one trial, OR = 2.07, 95% CI = 1.23 to 3.50, *p* = 0.006 for 12 months) (Figure S2). The focused ESWT group also had a better success rate than the control group at 3–6 and 12 months follow-ups (fixed-effect model, two trials, OR = 2.42, 95% CI = 1.14 to 5.13, *p* = 0.02 for three–six months; fixed-effect model, one trial, OR = 4.83, 95% CI = 1.21 to 19.22, *p* = 0.03 for 12 months) (Figure S3).

##### VAS Score

For the medium-energy level, one article used radial ESWT to treat plantar fasciitis and one article used focused ESWT. The radial ESWT group had better improvement in VAS pain scores than the control group at 6 and 12 months follow-ups (fixed-effect model, one trial, MD = −2.60, 95% CI = −3.78 to −1.42, *p* < 0.0001 for six-month; fixed-effect model, one trial, MD = −2.90, 95% CI = −3.98 to −1.82, *p* < 0.00001 for 12 months) (Figure S4). The focused ESWT group also had better improvement in VAS pain scores than the control group at 6 and 12 months follow-ups (fixed-effect model, one trial, MD = −6.88, 95% CI = −7.08 to −6.68, *p* < 0.00001 for six-month; fixed-effect model, one trial, MD = −4.56, 95% CI = −4.86 to −4.26, *p* < 0.00001 for 12-month) (Figure S5).

#### 3.3.3. Local Anesthesia

##### Success Rate

In the treatment with plantar fasciitis using high-energy focused ESWT, two articles did not use local anesthesia and three used local anesthesia. The pooled data showed no significant heterogeneity (*p*-value of chi-square >0.05 and *I*^2^ = 0%) (Figure S6). When they did not use local anesthesia, the ESWT group had a better success rate than the control groups at three-month follow-up (fixed-effect model, two trials, OR = 2.05, 95% CI = 1.28 to 3.28, *p* = 0.003) (Figure S6a). When they used local anesthesia, the ESWT group also had a better success rate than the control group at three-month follow-up (fixed-effect model, three trials, OR = 2.31, 95% CI = 1.62 to 3.29, *p* < 0.00001) (Figure S6b).

##### VAS Score

In the treatment with plantar fasciitis using high-energy focused ESWT, one article did not use local anesthesia and two used local anesthesia. When they did not use local anesthesia, the ESWT group had better improvement in VAS pain score than the control group at all the follow-ups (fixed-effect model, one trial, MD = −4.90, 95% CI = −5.53 to −4.27, *p* < 0.00001 for one-month; fixed-effect model, one trial, MD = −6.40, 95% CI = −7.01 to −5.79, *p* < 0.00001 for three-month) (Figure S7a,b). When they used local anesthesia, there was no significant difference in VAS pain score between the ESWT and control groups at one- and three-month follow-ups (fixed-effect model, one trial, MD = −0.40, 95% CI = −1.40 to 0.60, *p* = 0.43 for one-month; fixed-effect model, two trials, MD = −0.70, 95% CI = −1.41 to 0.01, *p* = 0.05 for three-month) (Figure S7c,d).

## 4. Discussion

ESWT is usually adopted to treat plantar fasciitis, and its energy level has been adjusted to achieve the maximum efficacy. Controversy remains regarding the efficacy of the energy levels used in ESWT in the treatment of plantar fasciitis. To clear the efficacy among different energy levels, randomized placebo-controlled trials were collected by systematic review, and the pooled data were estimated by meta-analysis. The results showed that the high-energy ESWT group had a better success rate than the control group only at three-month follow-up, but no significant difference between groups was observed for the other follow-up visit (one-month). In addition, no significant differences in VAS scores between the high-energy ESWT and control groups were observed for all the follow-up visits (one and three months). On the contrary, the medium-energy ESWT group had significantly better success rates than the control group for all the follow-up visits (3, 6, and 12 months). In addition, the medium-energy ESWT group had significant improvement in VAS scores compared with the control group for all the follow-up visits (1, 3, 6, and 12 months) after removing the extreme values. The low-energy ESWT group showed no significant difference in success rates but had significant improvement in VAS scores compared with the control group for all the follow-up visits. Under the medium-energy, the efficacy of radial and focused ESWTs was better than the control group. In the treatment of plantar fasciitis using high-energy focused ESWT, use of local anesthesia seems to reduce the efficacy of ESWT based on VAS scores. These results indicate that medium-energy ESWT in the treatment of plantar fasciitis was an effective treatment strategy in reducing pain, but the efficacy of low- and high-energy ESWTs was uncertain when compared with the control group.

To the best of our knowledge, the efficacy of ESWT in the treatment of plantar fasciitis at different follow-up times was firstly investigated using a systematic review and meta-analysis. The duration of efficacy varies with treatments. Some treatments show short-term effectiveness [40,41] and some show long-term effectiveness [42,43]. Thus, it is critical to understand the duration of efficacy of ESWT in treating plantar fasciitis. The current evidence showed that the medium-energy ESWT group was more effective than the control group for up to 12 months based on treatment success rates and VAS scores. However, the efficacy of low- and high-energy ESWTs was uncertain compared with the control group, probably because of limited number of included articles and lack of longer follow-up periods. More research for low- and high-energy ESWTs including longer follow-up periods is required to confirm the efficacy of these two energy levels in the treatment of plantar fasciitis in future studies.

Some trials adopted radial shockwaves, but some adopted focused waves. These two types of shockwaves have dramatic differences in physical characteristics. The efficacy of the two types of shockwaves in the treatment of plantar fasciitis has been reported to be different using meta-analysis [15]. They concluded that the efficacy of radial ESWT was better than that of focused ESWT with any energy levels. However, their comparisons were not under the same condition. To clear the difference in clinical outcomes between radial and focused ESWTs, we compared the two types of ESWT under the same condition, including the similar energy level (medium-energy) and the similar follow-up periods. The results showed that the efficacy of both radial and focused ESWT groups was better than the control group (Figures S2–S5). The efficacy of focused ESWT seems to be better than radial ESWT based on total OR and MD values in the meta-analysis. However, a limited number of articles were adopted in our subgroup analysis, and the results should be further confirmed.

The magnitude of pain increases with energy flux density because of local swelling and tenderness caused by shockwaves. In the past, patients usually required local anesthesia when receiving high-energy ESWT. However, local anesthesia can reduce the efficacy when using low-energy ESWT in the treatment of plantar fasciitis [21,44]. Among the seven included articles that used high-energy ESWT, three reported that they used local anesthesia when using ESWT (Table 1). All the included articles that used medium-energy ESWT did not use local anesthesia. Among two articles that used low-energy ESWT, one did not use local anesthesia. We made an attempt to investigate the effect of local anesthesia on clinical outcomes using meta-analysis. Our results show that use of local anesthesia seems to reduce the efficacy of high-energy focused ESWT based on VAS score. This could explain why the low-energy ESWT group with use of local anesthesia was not more effective than the control group, but low-energy ESWT group without use of local anesthesia was more effective. However, only one article of low-energy group was included in the subgroup analysis, and the efficacy of low-energy ESWT should be further confirmed.

A few meta-analyses have investigated the efficacy of different energy levels used in ESWT in the treatment of plantar fasciitis [18,19,20]. Medium- and high-energy ESWTs were reported to be effective in treating plantar fasciitis [19]. However, the number of trials adopted in the subgroup analysis was relatively small (one or two trials only) and it was difficult to draw a convincing conclusion. A previous study reported that the efficacy of low- and medium-energy ESWTs was superior over that of high-energy ESWT [18]. However, they did not investigate the efficacy at different follow-up periods and consider the effect of use of local anesthesia on the efficacy of ESWT. Our results showed use of local anesthesia could reduce the efficacy of ESWT. A previous meta-analysis showed that high-energy ESWT was effective in treating plantar fasciitis [8]. They only used the data at three-month follow-up for meta-analysis. Our results also show that high-energy ESWT group was more effective than the control group only at three-month follow-up based on treatment success rates. Our meta-analysis provided more detailed information about using ESWT in the treatment of plantar fasciitis.

The present meta-analysis has some limitations that might affect our conclusions. To investigate the efficacy of ESWT in the treatment of plantar fasciitis at different follow-ups, these included articles were divided into subgroups for meta-analysis. Some subgroups had relatively smaller number of trials (one or two trials) that could not draw convincing conclusions. To increase the number of trials for meta-analysis, we included all randomized placebo-controlled trials. Because three of the 14 included articles were not double-blinded, we could not perform a high-quality meta-analysis to prove the efficacy of ESWT using different energy levels. The treatment protocols using ESWT were varied among trials. Numbers of shocks and treatment duration that were adopted were varied among the trials. These different treatment protocols could have affected the results of our meta-analysis and should be further investigated.

## 5. Conclusions

Some factors that may affect the efficacy of ESWT have not been considered in the previous meta-analyses. In this study, we investigated the efficacy of ESWT with different energy levels in the treatment of plantar fasciitis at different follow-ups. The results show that medium-energy ESWT regardless of the types of shockwave generators was more effective for up to 12 months compared with the control group. However, the efficacy of low- and high-energy ESWTs was were uncertain compared with the control group because the results derived from treatment success rates and VAS scores were not consistent. We discovered that use of local anesthesia can reduce the efficacy of low- and high-energy ESWT. However, a limited number of articles in subgroup analysis and the lack of longer follow-up periods increased uncertainty about the efficacy of low- and high-energy ESWTs in our meta-analysis. More research is required to confirm the efficacy of low- and high-energy ESWTs at longer follow-up periods in the future. Under the medium-energy level, focused ESWT seems to be more effective than radial ESWT. Our results suggested that medium-energy ESWT without use of local anesthesia was more effective in treating plantar fasciitis for up to 12 months than the control group.

## Figures and Tables

**Table 1:** Main characteristics of the 14 included articles.

OBS	Study	Study Design	Treatment (Energy)	Use of Local Anesthesia	Number of Patients	Mean Age (Year)	Intensity (mJ/mm^2^)	Follow-Up Times	Extracted Outcome Data	Definition of Success
1	Rompe et al. 1996	NA, RCT	FSW (low)	No	50	44.0	0.08	3 and 13 months	VAS	
			Placebo		50	49.0				
2	Rompe et al. 2003	SB, RCT	FSW (medium)	No	22	43.0	0.16	6 and 12 months	Success rate and VAS	>50% improvement of pain during the first few minutes of walking scored on VAS
			Placebo		23	40.0				
3	Speed et al. 2003	DB, RCT	FSW (medium)	No	46	51.7	0.12	1, 2, 3, and 6 months	VAS	
			Placebo		42	52.5				
4	Haake et al. 2003	DB, RCT	SW (low)	Yes	129	NA	0.08	6 weeks, 3 months, and 12 months	Success rate	Roles and Maudsley score 1 or 2
			Placebo		132	NA				
5	Ogden et al. 2004	DB, RCT	FSW (high)	Yes	144	NA	0.22	3 months	Success rate	>50% improvement of pain scored on VAS and VAS of <=4
			Placebo		141	NA				
6	Theodore et al. 2004	DB, RCT	FSW (high)	Yes	73	50.0	0.36	6 weeks and 3 months	Success rate and VAS	Roles and Maudsley score 1 or 2
			Placebo		73	53.0				
7	Kudo et al. 2006	DB, RCT	FSW (high)	Yes	53	51.1	0.64	3 months	Success rate and VAS	>60% improvement of pain during the first few minutes of walking scored on VAS
			Placebo		52	48.8				
8	Gollwitzer et al. 2007	DB, RCT	FSW (high)	No	20	53.9	0.25	3 months	Success rate and VAS	Roles and Maudsley score 1 or 2
			Placebo		20	58.9				
9	Gerdesmeyer et al. 2008	DB, RCT	RSW (medium)	No	123	52.4	0.16	3 and 12 months	Success rate	>60% from baseline at follow-up after treatment for at least 2 of the 3 heel pain (VAS) measurements
			Placebo		116	52.0				
10	Marks et al. 2008	DB, RCT	RSW (medium)	No	16	51.9	0.16	6 months	Success rate	>50% improvement of pain scored on VAS
			Placebo		9	51.7				
11	Gollwitzer et al. 2015	DB, RCT	FSW (high)	No	125	50.0	0.25	3 months	Success rate	>60% from baseline at follow-up after treatment for at least 2 of the 3 heel pain (VAS) measurements.
			Placebo		121	47.4				
12	Hawamdeh et al. 2016	SB, RCT	RSW (high)	No	12	NA	0.25	3 weeks	VAS	
			Placebo		12	NA				
13	Ibrahim et al. 2017	DB, RCT	RSW (medium)	No	25	56.6	0.16	1, 3, 6, and 13 months	VAS	
			Placebo		25	49.1				
14	Takla et al. 2019	SB, RCT	FSW (high)	No	30	NA	0.22–0.28	3 weeks and 3 months	VAS	
			Placebo		30	NA				

NA: not available; SB: single-blind; DB: double-blind; RCT: randomized controlled trail; RSW: radial shockwave; FSW: focused shockwave; VAS: visual analog.

## Supplementary Materials

The following are available online at https://www.mdpi.com/2077-0383/8/9/1497/s1.

## Appendix A

**Table:**

Section/Topic	#	Checklist Item	Reported on Page #
**TITLE**			
Title	1	Identify the report as a systematic review, meta-analysis, or both.	1
ABSTRACT			
Structured summary	2	Provide a structured summary including, as applicable: background; objectives; data sources; study eligibility criteria, participants, and interventions; study appraisal and synthesis methods; results; limitations; conclusions and implications of key findings; systematic review registration number.	1
**INTRODUCTION**			
Rationale	3	Describe the rationale for the review in the context of what is already known.	2
Objectives	4	Provide an explicit statement of questions being addressed with reference to participants, interventions, comparisons, outcomes, and study design (PICOS).	2
**METHODS**			
Protocol and registration	5	Indicate if a review protocol exists, if and where it can be accessed (e.g., Web address), and, if available, provide registration information including registration number.	N/A
Eligibility criteria	6	Specify study characteristics (e.g., PICOS, length of follow-up) and report characteristics (e.g., years considered, language, publication status) used as criteria for eligibility, giving rationale.	3
Information sources	7	Describe all information sources (e.g., databases with dates of coverage, contact with study authors to identify additional studies) in the search and date last searched.	3
Search	8	Present full electronic search strategy for at least one database, including any limits used, such that it could be repeated.	3
Study selection	9	State the process for selecting studies (i.e., screening, eligibility, included in systematic review, and, if applicable, included in the meta-analysis).	3
Data collection process	10	Describe method of data extraction from reports (e.g., piloted forms, independently, in duplicate) and any processes for obtaining and confirming data from investigators.	3
Data items	11	List and define all variables for which data were sought (e.g., PICOS, funding sources) and any assumptions and simplifications made.	3
Risk of bias in individual studies	12	Describe methods used for assessing risk of bias of individual studies (including specification of whether this was done at the study or outcome level), and how this information is to be used in any data synthesis.	3–4
Summary measures	13	State the principal summary measures (e.g., risk ratio, difference in means).	4
Synthesis of results	14	Describe the methods of handling data and combining results of studies, if done, including measures of consistency (e.g., I^2^) for each meta-analysis.	4
Risk of bias across studies	15	Specify any assessment of risk of bias that may affect the cumulative evidence (e.g., publication bias, selective reporting within studies).	3-4
Additional analyses	16	Describe methods of additional analyses (e.g., sensitivity or subgroup analyses, meta-regression), if done, indicating which were pre-specified.	4
**RESULTS**			
Study selection	17	Give numbers of studies screened, assessed for eligibility, and included in the review, with reasons for exclusions at each stage, ideally with a flow diagram.	Page 4, Figure 1
Study characteristics	18	For each study, present characteristics for which data were extracted (e.g., study size, PICOS, follow-up period) and provide the citations.	Page 4, Table 1
Risk of bias within studies	19	Present data on risk of bias of each study and, if available, any outcome level assessment (see item 12).	Figure 3
Results of individual studies	20	For all outcomes considered (benefits or harms), present, for each study: (a) simple summary data for each intervention group (b) effect estimates and confidence intervals, ideally with a forest plot.	Pages 9–13, Figure 4, Figure 5, Figure 6, Figure 7, Figure 8 and Figure 9
Synthesis of results	21	Present results of each meta-analysis done, including confidence intervals and measures of consistency.	9–13
Risk of bias across studies	22	Present results of any assessment of risk of bias across studies (see Item 15).	8
Additional analysis	23	Give results of additional analyses, if done (e.g., sensitivity or subgroup analyses, meta-regression (see Item [16]).	Table S1
**DISCUSSION**			
Summary of evidence	24	Summarize the main findings including the strength of evidence for each main outcome; consider their relevance to key groups (e.g., healthcare providers, users, and policy makers).	13–14
Limitations	25	Discuss limitations at study and outcome level (e.g., risk of bias), and at review-level (e.g., incomplete retrieval of identified research, reporting bias).	15
Conclusions	26	Provide a general interpretation of the results in the context of other evidence, and implications for future research.	15
**FUNDING**			
Funding	27	Describe sources of funding for the systematic review and other support (e.g., supply of data); role of funders for the systematic review.	N/A
