# Supplementary material for: Efficacy of Different Energy Levels Used in Focused and Radial Extracorporeal Shockwave Therapy in the Treatment of Plantar Fasciitis: A Meta-Analysis of Randomized Placebo-Controlled Trials
Source: J Clin Med. 2019 Sep 19;8(9):1497. doi: 10.3390/jcm8091497 (PMC6780733; doi:10.3390/jcm8091497)
Supplement: Supplementary file 1 [file jcm-08-01497-s001.pdf]

Supplement material

Table S1. A sensitivity analysis for meta-analysis of visual analog scale (VAS) scores in medium-energy ESWT and placebo-controlled groups.

Altered dataset	Follow-up time (month)	Model	Number of trials	Mean difference	95% CI	p-value	I^2
Removal of Speed et al. 2003	1	Fixed	1	-6.92	-7.12, -6.72	<0.00001	NA
Removal of Ibrahim et al. 2017	1	Fixed	1	-0.12	-1.06, 0.82	0.80	NA
Removal of Speed et al. 2003	3	Fixed	1	-6.64	-6.78, -6.50	<0.00001	NA
Removal of Ibrahim et al. 2017	3	Fixed	1	-0.57	-1.81, 0.67	0.37	NA
Removal of Speed et al. 2003	6	Random	2	-4.78	-8.97, -0.59	0.03	98%
Removal of Rompe et al. 2003	6	Random	2	-3.18	-10.49, 4.12	0.39	99%
Removal of Ibrahim et al. 2017	6	Random	2	-1.03	-4.14, 2.08	0.52	92%
Removal of Rompe et al. 2003	12	Fixed	1	-4.56	-4.86, -4.26	<0.00001	NA
Removal of Ibrahim et al. 2017	12	Fixed	1	-2.90	-3.98, -1.82	<0.00001	NA

NA : not avaliable

(a) 1-month follow-up

Study or Subgroup	ESWT Mean	ESWT SD	ESWT Total	Control Mean	Control SD	Control Total	Weight	Mean Difference IV, Fixed, 95% CI
Ibrahim et al. 2017	0.64	0.31	25	7.56	0.42	25	100.0%	-6.92 [-7.12, -6.72]
**Total (95% CI)**			**25**			**25**	**100.0%**	**-6.92 [-7.12, -6.72]**

Heterogeneity: Not applicable
Test for overall effect: Z = 66.28 (P < 0.00001)

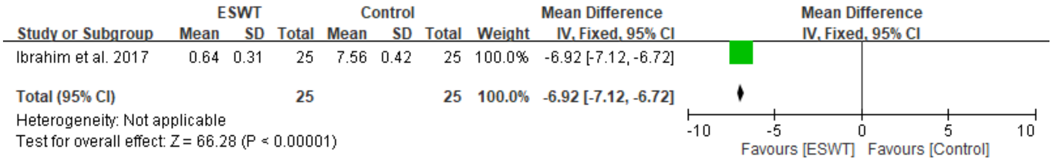

(b) 3-month follow-up

Study or Subgroup	ESWT Mean	ESWT SD	ESWT Total	Control Mean	Control SD	Control Total	Weight	Mean Difference IV, Fixed, 95% CI
Ibrahim et al. 2017	1.08	0.28	25	7.72	0.24	25	100.0%	-6.64 [-6.78, -6.50]
**Total (95% CI)**			**25**			**25**	**100.0%**	**-6.64 [-6.78, -6.50]**

Heterogeneity: Not applicable
Test for overall effect: Z = 90.03 (P < 0.00001)

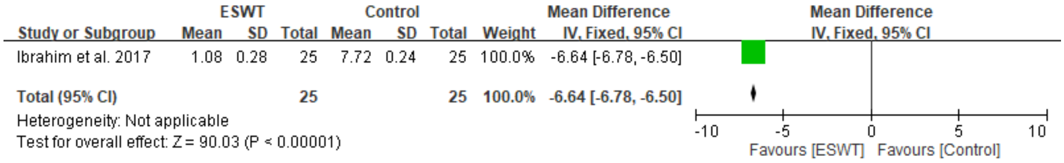

(c) 6-month follow-up

Study or Subgroup	ESWT Mean	ESWT SD	ESWT Total	Control Mean	Control SD	Control Total	Weight	Mean Difference IV, Random, 95% CI
Rompe et al. 2003	2.1	2	20	4.7	1.9	22	49.0%	-2.60 [-3.78, -1.42]
Ibrahim et al. 2017	0.52	0.14	25	7.4	0.49	25	51.0%	-6.88 [-7.08, -6.68]
**Total (95% CI)**			**45**			**47**	**100.0%**	**-4.78 [-8.97, -0.59]**

Heterogeneity: $Tau^2 = 8.97$; $Chi^2 = 48.92$, df = 1 ($P < 0.00001$); $I^2 = 98\%$
Test for overall effect: Z = 2.23 (P = 0.03)

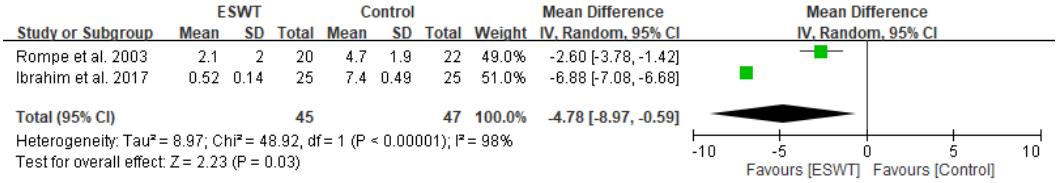

Supplement Fig. 1. Forest plots of visual analog scale (VAS) scores in medium-energy ESWT and placebo-controlled groups at 1-month (a), 3-month (b), and 6-month (c) follow-ups

(a) Radial ESWT at 3-6 months follow-up

Study or Subgroup	ESWT Events	ESWT Total	Control Events	Control Total	Weight	Odds Ratio M-H, Fixed, 95% CI
Gerdesmeyer et al. 2008	77	123	49	116	89.4%	2.29 [1.36, 3.85]
Marks et al. 2008	9	16	4	9	10.6%	1.61 [0.31, 8.32]
**Total (95% CI)**		**139**		**125**	**100.0%**	**2.22 [1.35, 3.63]**
Total events	86		53			

Heterogeneity: Chi² = 0.16, df = 1 (P = 0.69); I² = 0%
Test for overall effect: Z = 3.16 (P = 0.002)

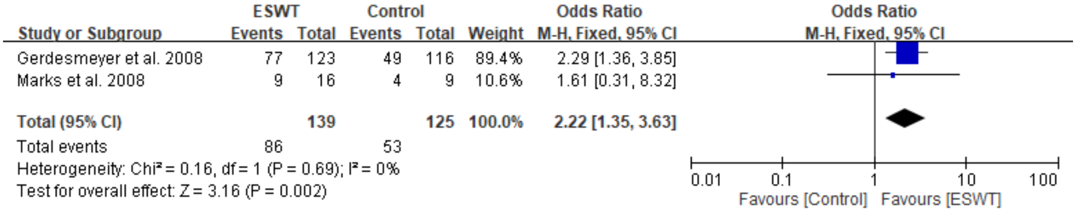

(b) Radial ESWT at 12-month follow-up

Study or Subgroup	ESWT Events	ESWT Total	Control Events	Control Total	Weight	Odds Ratio M-H, Fixed, 95% CI
Gerdesmeyer et al. 2008	78	123	51	112	100.0%	2.07 [1.23, 3.50]
**Total (95% CI)**		**123**		**112**	**100.0%**	**2.07 [1.23, 3.50]**
Total events	78		51			

Heterogeneity: Not applicable
Test for overall effect: Z = 2.74 (P = 0.006)

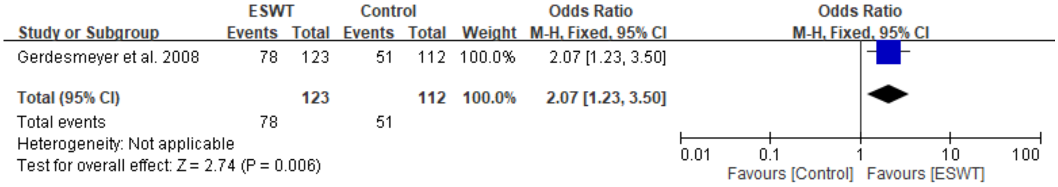

Supplement Fig. 2. Forest plots of treatment success rates in medium-energy radial ESWT placebo-controlled groups at 3-6-month (a) and 12-month (b) follow-ups.

(a) Focused ESWT at 3-6 months follow-up

Study or Subgroup	ESWT Events	ESWT Total	Control Events	Control Total	Weight	Odds Ratio M-H, Fixed, 95% CI
Speed et al. 2003	17	46	10	42	74.3%	1.88 [0.74, 4.75]
Rompe et al. 2003	12	20	6	22	25.7%	4.00 [1.09, 14.62]
**Total (95% CI)**		**66**		**64**	**100.0%**	**2.42 [1.14, 5.13]**
Total events	29		16			

Heterogeneity: Chi² = 0.87, df = 1 (P = 0.35); I² = 0%
Test for overall effect: Z = 2.31 (P = 0.02)

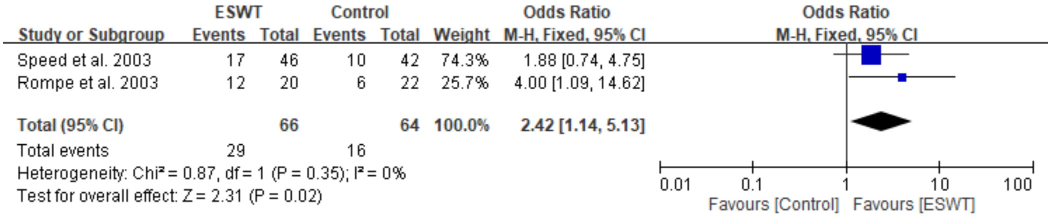

(b) Focused ESWT at 12 months follow-up

Study or Subgroup	ESWT Events	ESWT Total	Control Events	Control Total	Weight	Odds Ratio M-H, Fixed, 95% CI
Rompe et al. 2003	13	18	7	20	100.0%	4.83 [1.21, 19.22]
**Total (95% CI)**		**18**		**20**	**100.0%**	**4.83 [1.21, 19.22]**
Total events	13		7			

Heterogeneity: Not applicable
Test for overall effect: Z = 2.23 (P = 0.03)

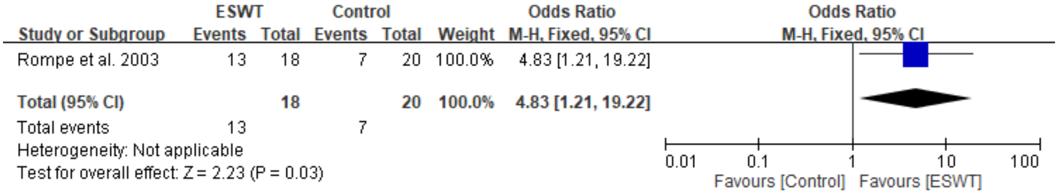

Supplement Fig. 3. Forest plots of treatment success rates in medium-energy focused ESWT and placebo-controlled groups at 3-6-month (a) and 12-month (b) follow-ups.

(a) Radial ESWT at 6 months follow-up

Study or Subgroup	ESWT Mean	ESWT SD	ESWT Total	Control Mean	Control SD	Control Total	Weight	Mean Difference IV, Fixed, 95% CI
Rompe et al. 2003	2.1	2	20	4.7	1.9	22	100.0%	-2.60 [-3.78, -1.42]
**Total (95% CI)**			**20**			**22**	**100.0%**	**-2.60 [-3.78, -1.42]**

Heterogeneity: Not applicable
Test for overall effect: Z = 4.31 (P < 0.0001)

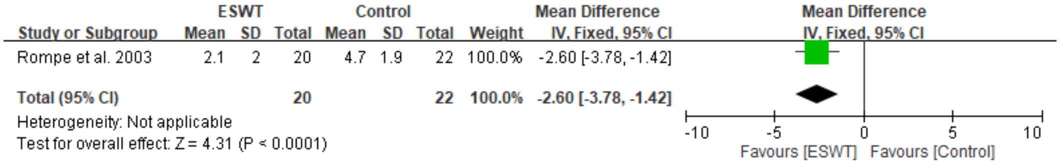

(b) Radial ESWT at 12 months follow-up

Study or Subgroup	ESWT Mean	ESWT SD	ESWT Total	Control Mean	Control SD	Control Total	Weight	Mean Difference IV, Fixed, 95% CI
Rompe et al. 2003	1.5	1.7	18	4.4	1.7	20	100.0%	-2.90 [-3.98, -1.82]
**Total (95% CI)**			**18**			**20**	**100.0%**	**-2.90 [-3.98, -1.82]**

Heterogeneity: Not applicable
Test for overall effect: Z = 5.25 (P < 0.00001)

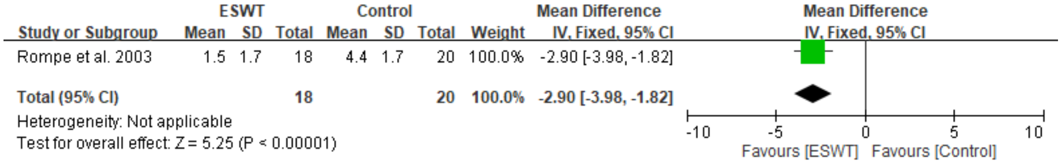

Supplement Fig. 4. Forest plots of visual analog scale (VAS) scores in medium-energy radial ESWT and placebo-controlled groups at 6-month (a) and 12-month (b) follow-ups.

(a) Focused ESWT at 6 months follow-up

Study or Subgroup	ESWT Mean	ESWT SD	ESWT Total	Control Mean	Control SD	Control Total	Weight	Mean Difference IV, Fixed, 95% CI
Ibrahim et al. 2017	0.52	0.14	25	7.4	0.49	25	100.0%	-6.88 [-7.08, -6.68]
**Total (95% CI)**			**25**			**25**	**100.0%**	**-6.88 [-7.08, -6.68]**

Heterogeneity: Not applicable
Test for overall effect: Z = 67.50 (P < 0.00001)

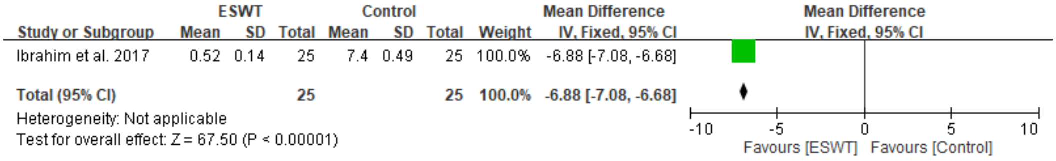

(b) Focused ESWT at 12 months follow-up

Study or Subgroup	ESWT Mean	ESWT SD	ESWT Total	Control Mean	Control SD	Control Total	Weight	Mean Difference IV, Fixed, 95% CI
Ibrahim et al. 2017	2.32	0.43	25	6.88	0.64	25	100.0%	-4.56 [-4.86, -4.26]
**Total (95% CI)**			**25**			**25**	**100.0%**	**-4.56 [-4.86, -4.26]**

Heterogeneity: Not applicable
Test for overall effect: Z = 29.57 (P < 0.00001)

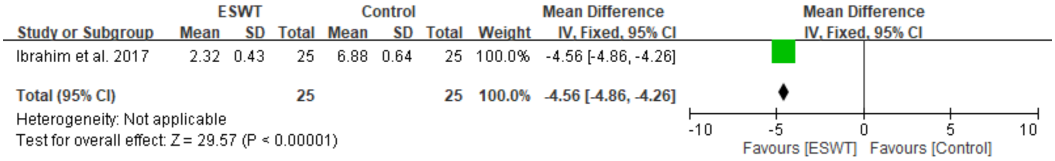

Supplement Fig. 5. Forest plots of visual analog scale (VAS) scores in medium-energy focused ESWT and placebo-controlled groups at 6-month (a) and 12-month (b) follow-ups.

(a) without use of local anesthesia

Study or Subgroup	ESWT Events	ESWT Total	Control Events	Control Total	Weight	Odds Ratio M-H, Fixed, 95% CI
Gollwitzer et al. 2007	12	20	8	20	13.3%	2.25 [0.63, 7.97]
Gollwitzer et al. 2015	68	125	45	121	86.7%	2.01 [1.21, 3.35]
**Total (95% CI)**		**145**		**141**	**100.0%**	**2.05 [1.28, 3.28]**
Total events	80		53			

Heterogeneity: Chi² = 0.03, df = 1 (P = 0.87); I² = 0%
Test for overall effect: Z = 2.97 (P = 0.003)

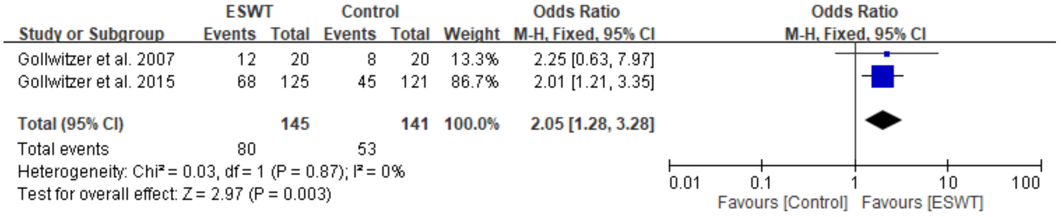

(b) with use of local anesthesia

Study or Subgroup	ESWT Events	ESWT Total	Control Events	Control Total	Weight	Odds Ratio M-H, Fixed, 95% CI
Theodore et al. 2004	45	73	29	73	27.7%	2.44 [1.25, 4.74]
Ogden et al. 2004	67	144	42	141	56.4%	2.05 [1.26, 3.34]
Kudo et al. 2006	25	53	12	52	15.9%	2.98 [1.28, 6.90]
**Total (95% CI)**		**270**		**266**	**100.0%**	**2.31 [1.62, 3.29]**
Total events	137		83			

Heterogeneity: Chi² = 0.60, df = 2 (P = 0.74); I² = 0%
Test for overall effect: Z = 4.60 (P < 0.00001)

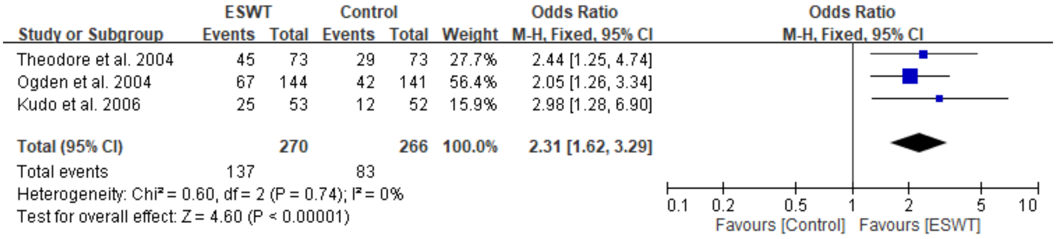

Supplement Fig. 6. Forest plots of treatment success rates in high-energy ESWT without (a) and with (b) use of local anesthesia and placebo-controlled groups at 3-month follow-up.

(a) without use of local anesthesia at 1-month follow-up

Study or Subgroup	ESWT Mean	ESWT SD	ESWT Total	Control Mean	Control SD	Control Total	Weight	Mean Difference IV, Fixed, 95% CI
Takla et al. 2019	3.3	1.2	30	8.2	1.3	30	100.0%	-4.90 [-5.53, -4.27]
**Total (95% CI)**			**30**			**30**	**100.0%**	**-4.90 [-5.53, -4.27]**

Heterogeneity: Not applicable
Test for overall effect: Z = 15.17 (P < 0.00001)

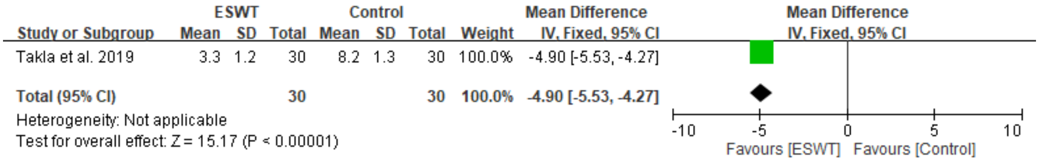

(b) without use of local anesthesia at 3-month follow-up

Study or Subgroup	ESWT Mean	ESWT SD	ESWT Total	Control Mean	Control SD	Control Total	Weight	Mean Difference IV, Fixed, 95% CI
Takla et al. 2019	1.7	1.02	27	8.1	1.2	25	100.0%	-6.40 [-7.01, -5.79]
**Total (95% CI)**			**27**			**25**	**100.0%**	**-6.40 [-7.01, -5.79]**

Heterogeneity: Not applicable
Test for overall effect: Z = 20.64 (P < 0.00001)

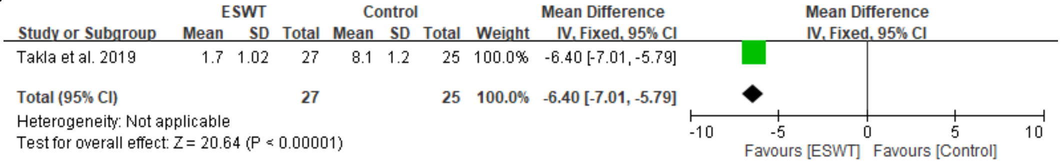

(c) with use of local anesthesia at 1-month follow-up

Study or Subgroup	ESWT Mean	ESWT SD	ESWT Total	Control Mean	Control SD	Control Total	Weight	Mean Difference IV, Fixed, 95% CI
Theodore et al. 2004	4.6	3.1	72	5	3	71	100.0%	-0.40 [-1.40, 0.60]
**Total (95% CI)**			**72**			**71**	**100.0%**	**-0.40 [-1.40, 0.60]**

Heterogeneity: Not applicable
Test for overall effect: Z = 0.78 (P = 0.43)

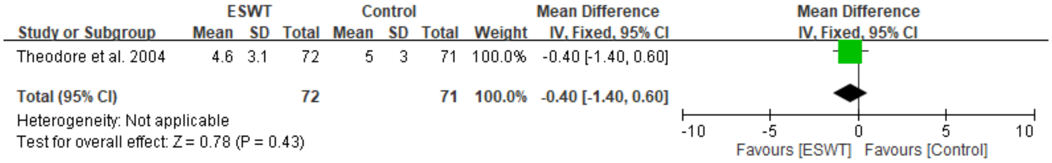

(d) with use of local anesthesia at 3-month follow-up

Study or Subgroup	ESWT Mean	ESWT SD	ESWT Total	Control Mean	Control SD	Control Total	Weight	Mean Difference IV, Fixed, 95% CI
Theodore et al. 2004	3.4	2.7	73	4.1	3.1	73	56.6%	-0.70 [-1.64, 0.24]
Kudo et al. 2006	3.7	3.1	53	4.4	2.5	52	43.4%	-0.70 [-1.78, 0.38]
**Total (95% CI)**			**126**			**125**	**100.0%**	**-0.70 [-1.41, 0.01]**

Heterogeneity: Chi² = 0.00, df = 1 (P = 1.00); I² = 0%
Test for overall effect: Z = 1.93 (P = 0.05)

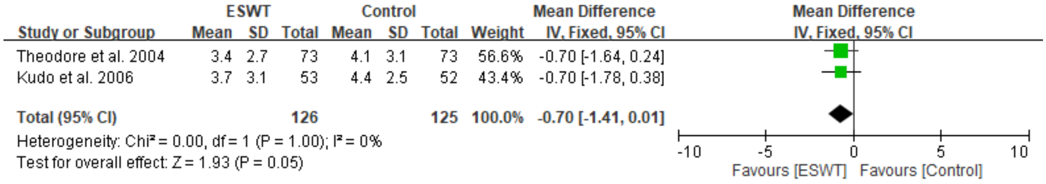

Supplement Fig. 7. Forest plots of visual analog scale (VAS) scores in high-energy ESWT without use of local anesthesia at 1-month (a) and 3-month (b) follow-ups and with use of local anesthesia at 1-month (c) and 3-month (d) follow-ups.
